# The Application of Natural Phenolic Substances as Antimicrobial Agents in Agriculture and Food Industry

**DOI:** 10.3390/foods14111893

**Published:** 2025-05-26

**Authors:** Katarzyna Dembińska, Ambika H. Shinde, Marcela Pejchalová, Agnieszka Richert, Maria Swiontek Brzezinska

**Affiliations:** 1 Department of Environmental Microbiology and Biotechnology, Faculty of Biological and Veterinary Sciences, Nicolaus Copernicus University in Torun, Lwowska 1, 87-100 Toruń, Poland; ambikahshinde@gmail.com (A.H.S.); swiontek@umk.pl (M.S.B.); 2 Department of Biological and Biochemical Sciences, Faculty of Chemical Technology, University of Pardubice, Sudentska 573, 53210 Pardubice, Czech Republic; marcela.pejchalova@upce.cz; 3 Department of Genetics, Faculty of Biological and Veterinary Sciences, Nicolaus Copernicus University in Torun, Lwowska 1, 87-100 Toruń, Poland; a.richert@umk.pl

**Keywords:** natural phenolic compounds, antimicrobial agents, food preservation, agriculture, food safety

## Abstract

Natural phenolic substances have emerged as promising alternatives to synthetic antimicrobials in both agriculture and the food industry, where concerns over microbial resistance and chemical residues are rising. This review provides a comprehensive overview of the current literature, highlighting the potential of these compounds as effective antimicrobial agents. A systematic evaluation of in vitro and in vivo studies was conducted, focusing on the efficacy of various phenolic compounds against a range of pathogens. The analysis revealed that natural phenolics not only inhibit microbial growth but also enhance the shelf life and safety of food products and protect crops from disease. Moreover, although laboratory results are promising, the translation of these findings into practical applications requires further investigation. Overall, the evidence supports the potential for natural phenolic substances to serve as integral components in sustainable agriculture and food preservation strategies.

## 1. Introduction

Plant-derived substances are gaining research interest due to their antimicrobial properties. For centuries, herbs and spices have been used for food preservation due to their bioactive substances. Modern studies indicate that purified plant-derived components often exhibit stronger antimicrobial activity than when included in essential oils [1]. Phenolic compounds, abundant plant secondary metabolites, play a key role in plant protection and can also impact fruit and vegetable coloration [2]. Due to their antimicrobial and antioxidant properties, they are being explored for applications in agriculture, food packaging, cosmetics, and medicine.

A major challenge in agriculture is ensuring sustainability while maintaining productivity. The EU’s Green Deal aims to cut chemical plant protection product use by 50% by 2030 [3]. Conventional methods pose environmental and health risks [4], prompting research into sustainable alternatives. One promising approach is nanomaterial-based carriers for plant protection agents [5]. Another, discussed in this review, is the use of phenolic compounds as biopesticides against fungal pathogens, which cause 70–80% of crop losses [6].

Concerns over synthetic preservatives in food have fueled demand for natural alternatives. While preservatives are essential for food safety, phenolic compounds can be incorporated into packaging materials to extend shelf life. This can be achieved by direct incorporation into the polymer matrix, antimicrobial sachets, or coatings with immobilized biocides [7].

This review aims to detail the most commonly used natural phenolic compounds in agriculture and the food industry.

## 2. Classification of Phenolic Compounds

Phenolic compounds, characterized by a hydroxyl group attached to an aromatic ring, are secondary metabolites produced in response to stress. They contribute to pigmentation and protection against UV radiation, pathogens, and herbivores [8]. Based on their structural characteristics, they are classified into two main groups: flavonoid and non-flavonoid compounds. The non-flavonoid group includes phenolic acids (hydroxybenzoic and hydroxycinnamic acids), stilbenes, coumarins, tannins, monoterpenoid phenols, and phenylpropanoids. The flavonoid group consists of flavonols, flavones, flavan-3-ols, flavanones, isoflavones, and anthocyanidins, which differ in the oxidation state of the central C-ring and the presence of hydroxyl or glycosyl groups [9].

Table 1 shows selected representatives of these structural classes, along with their chemical names and natural sources. These compounds are commonly found in plant-derived materials, including fruits, vegetables, herbs, spices, tea, and wine. Due to their diverse biological activities, particularly antimicrobial properties, they are of increasing interest for applications in agriculture and food preservation.

## 3. The Biocidal Mechanism of Action of Phenolic Compounds

Phenolic compounds exhibit antimicrobial activity through multiple mechanisms that disrupt microbial cell structures and metabolic processes. Their biocidal effects depend on structural features, such as the number and position of hydroxyl groups, molecular size, and lipophilicity.

One of the primary mechanisms involves cell membrane disruption. Phenolic compounds interact with lipid bilayers, increasing membrane permeability and causing leakage of intracellular components, ultimately leading to cell lysis. Lipophilic compounds, such as thymol, carvacrol, cinnamaldehyde, and eugenol, insert into the membrane, altering its fluidity and integrity [24].

Another crucial mechanism is protein denaturation and enzyme inhibition. Phenolics form hydrogen bonds with proteins, disrupting enzymatic functions essential for microbial survival. For example, flavonoids like quercetin and catechins inhibit ATP synthase, reducing energy production [25]. Additionally, phenolic compounds induce oxidative stress by generating reactive oxygen species (ROS) [26]. This leads to DNA damage, lipid peroxidation, and protein oxidation, which contribute to microbial cell death. Finally, phenolic compounds can interfere with quorum sensing and biofilm formation, reducing microbial virulence. Certain flavonoids and phenolic acids inhibit bacterial communication systems, preventing coordinated behaviors essential for infection and persistence [27].

The antimicrobial activity of phenolic compounds is strongly influenced by their molecular structure. The presence of hydroxyl groups (-OH) allows substances to interact with charged molecules such as RNA, DNA, or proteins, thus triggering an antimicrobial effect. The presence of phenyl groups (-C_5_H_9_), methoxyl groups (-OCH_3_), and glycosylation is also important [28]. Studies also indicate that combinations of some phenolic compounds can act synergistically, such as cinnamaldehyde and citronellal [29] as well as thymol and carvacrol [30].

**Figure 1:**
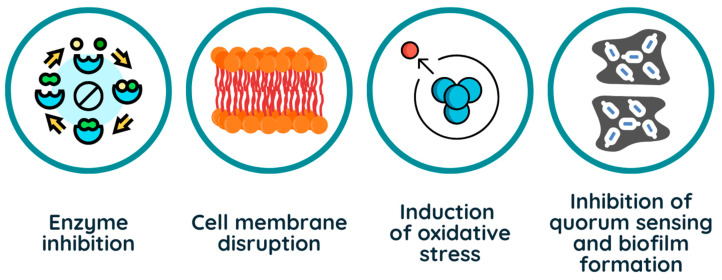
Main mechanisms of antimicrobial activity of phenolic compounds.

Overall, the biocidal activity of phenolic compounds results from a combination of membrane disruption, enzyme inhibition, oxidative damage, and anti-quorum sensing effects (Figure 1), making them promising antimicrobial agents in food preservation and agriculture.

Despite their proven antimicrobial activity, many phenolic compounds are characterized by volatility and susceptibility to environmental degradation, which can limit their usefulness. For this reason, modern systems are being developed to increase bioavailability, allow controlled and prolonged release, and reduce doses of substances with the same antimicrobial effect. Among these systems, the following can be mentioned: nanoemulsions, liposomes, polymeric microcapsules, or lipid nanoparticles [31]. Another factor that may limit the activity of phenolic substances is their sensitivity to pH. Data in the literature indicate that they degrade rapidly under alkaline conditions through auto-oxidation processes. In a study by Pasquet et al. [32], gallic acid concentration decreased by 97% at pH = 13.5, while the compound remained stable at pH = 2. This effect was also confirmed in food products of different pH, where the content of phenolic compounds decreased in products with higher pH [33]. The stability of phenolic substances also depends on the matrix in which they are found. For example, in the presence of proteins, they have reduced stability and bioavailability, and in the presence of lipids, these properties are increased [34].

## 4. Phenolic Compounds with the Highest Application Potential

### 4.1. In Agriculture

In agriculture, phenolic compounds have emerged as promising agents for controlling a wide range of phytopathogens and food spoilage microorganisms. Some of the most common fungal plant pathogens are *Botrytis* spp., *Fusarium* spp., *Phytophthora* spp., or *Sclerotinia* spp., which are responsible for severe crop losses worldwide. For example, *Botrytis cinerea* causes gray mold in many crops, primarily attacking soft tissues like fruits, vegetables, and flowers. This species is phylogenetically related to *Sclerotinia sclerotiorum*, a more aggressive pathogen that causes white mold [35,36]. Most crops are susceptible to fungal diseases, including those of greatest economic importance like rice and wheat [37].

The antimicrobial potential of phenolic compounds has been extensively demonstrated through various in vitro and in vivo studies, highlighting their capacity to inhibit fungal growth, disrupt cell membranes, and interfere with toxin biosynthesis.

**Cinnamaldehyde (CN)** is one of the most studied compounds in this context. It has shown robust antifungal activity, effectively inhibiting the growth of *Aspergillus niger* at a minimum inhibitory concentration (MIC) of 40 µg/mL, with efficacy sustained for up to 14 days [38]. In addition, CN exerts a dose-dependent inhibitory effect on *Aspergillus flavus* by arresting radial growth, conidiation, and aflatoxin B1 production; a concentration of 104 mg/L completely halts toxin production by reducing oxidative stress and altering the redox balance [39]. At the cellular level, CN induces apoptosis in *A. flavus* through elevated calcium ion levels and reactive oxygen species (ROS) production, along with mitochondrial dysfunction and DNA damage [26]. Notably, its vapor phase has proven even more effective than the liquid form by impairing mitochondrial function and compromising plasma membrane integrity in *A. niger*, thereby delaying post-harvest degradation in stored grains [40].

Beyond these direct antifungal effects, CN also shows promise against other pathogenic molds. Its derivative, α-methyl-CN, demonstrates potent antifungal activity against *Fusarium oxysporum*, significantly inhibiting spore germination and reducing pathogenicity in vivo [41,42]. Moreover, CN has been reported to enhance the sensitivity of pathogens such as *F. oxysporum* (causing potato dry rot) to environmental stresses and to effectively control *Fusarium verticillioides*, a producer of fumonisin mycotoxins, with MIC values varying based on the source and formulation [43]. Its combination with other compounds, such as citronellal, eugenol, and carvacrol, further improves its antifungal efficacy against pathogens like *Penicillium digitatum* [29,44]. The combination of CN and citronellal in a 5:16 (*v*/*v*) ratio reduced the MIC against this pathogen to 0.40 mL/L, compared to 0.50 mL/L for pure CN and 1.60 mL/L for citronellal, indicating a synergistic effect [29].

Additionally, CN disrupts the metabolism of less common pathogens such as *Phytophthora capsici*, contributing to the extended shelf life of treated produce [45]. CN has also exhibited bioactivity against many horticulture-associated fungal pathogens and some other food-associated fungi like *Botryodiploida theobromae*, *Alternaria alternata*, *Candida albicans*, *Gliocephalotrichum microchlamydosporum*, and *Colletotrichum musae* [46,47].

Its antibacterial potential is also notable, effectively inhibiting foodborne pathogens such as various *Salmonella* serovars, *Pseudomonas syringae*, and *Xanthomonas citri*, with performance comparable to conventional disinfectants but without corrosive or toxic by-products [48,49]. Moreover, CN has been successfully employed as a fumigant against *Agrobacterium tumefaciens* and shows activity against other significant bacterial pathogens in agricultural settings, such as *Erwinia amylovora*, *Pectobacterium aroidearum*, *Pseudomonas aeruginosa*, and *Ralstonia solanacearum* [50,51].

CN has also been reported in various studies to possess antimicrobial activities against some other food-borne and plant as well as human pathogens in vitro in laboratory media, animal feeds, and human foods contaminated with disease-causing bacteria including *Streptococcus pyogenes*, *P. aeruginosa*, *Staphylococcus epidermidis*, *Staphylococcus pseudintermedius*, *Proteus mirabilis*, *Streptococcus mutans*, *Salmonella typhimurium*, *Bacillus cereus*, *Campylobacter jejuni*, *Clostridium perfringens*, *Listeria monocytogenes*, and *Salmonella enterica* [51,52].

However, the use of CN may be limited due to its high water solubility, high volatility, and susceptibility to degradation by environmental factors, which can result in reduced effectiveness over time. Therefore, various encapsulation techniques are being developed to enhance its stability and control its release. For example, cinnamaldehyde-loaded nonoethosomes at a concentration of 32.62 g/g ethosome have been shown to be stable at low pH (>4) and a temperature of 37 °C. Moreover, the MIC of the nanoethosomes was lower than that of free CN, amounting to 106.8 µg/mL for *Colletotrichum musae*, compared to 160.2 µg/mL for the free compound [53].

**Cinnamic acid (CA)** also exhibits considerable potential in agriculture, primarily by inhibiting fungal growth and enhancing plant defense mechanisms. CA has been shown to reduce mycelial growth and spore germination in pathogens like *S. sclerotiorum*, achieving up to 95% effectiveness in pot experiments on oilseed rape [54]. Derivatives of CA further disrupt the cellular integrity of pathogens such as *Gaeumannomyces graminis* var. *tritici*, *Valsa mali*, and *B. cinerea* by inducing ROS-mediated cell death [55]. At a concentration of 200 µg/mL, one of the derivatives studied by Yang et al. [56] also achieved 84.3% protective efficacy against *Xanthomonas axonopodis* pv. *citri*, while innovative formulations, such as chitosan–cinnamic acid conjugates, have demonstrated promising results against *R. solanacearum*, the agent of bacterial wilt [57]. These studies underscore CA’s dual role as both a direct antimicrobial and a plant defense enhancer.

**Carvacrol (CR)** is another compound with diverse agricultural applications, showing strong antifungal and antibacterial properties. Its efficacy against a variety of fungal pathogens has been well documented; for instance, CR demonstrates potent inhibitory effects on species such as *Cladosporium*, *Aspergillus*, *F. oxysporum*, *Penicillium*, *Rhizopus oryzae*, and *B. cinerea*, with MIC values ranging from 100 to 150 µg/mL [58]. In addition to its standalone effects, CR in combination with thymol and eugenol exerts synergistic action against *Fusarium* species, offering a natural alternative for controlling diseases like ray grass fusariosis [59].

Its mechanism of action involves membrane damage, leading to leakage of cytoplasmic contents—a mode of action that also benefits post-harvest preservation by extending the shelf life of fruits such as grapes [60]. Furthermore, CR has demonstrated promising antibacterial effects against pathogens such as *Xanthomonas* spp., suggesting its potential use as a seed disinfectant and in the management of bacterial spot disease in vegetables [61,62]. Its role in controlling damping-off disease in seedlings further highlights its broad application potential in agriculture [59]. CR also showed one of the strongest inhibitory effects on the mycelial growth of *Helminthosporium solani* that causes Potato silver scurf under in vitro conditions [63]. Carvacrol exhibits low sorption in soil and thus high bioavailability. On the other hand, it can also reduce the enzyme activity of native soil microorganisms [64].

**Resveratrol (RS)** has been widely studied for its antimicrobial properties against several bacteria and fungi of agricultural significance. It acts on bacterial wilt, caused by *R. solanacearum*, a pathogen widely distributed in tobacco crops worldwide. RS primarily disrupts bacterial cell membranes, inhibits swarming motility, and prevents biofilm formation. A pot experiment revealed that RS significantly reduced the early adhesion and colonization of *R. solanacearum* on tobacco plants, achieving a control efficiency of 85% after 13 days of incubation [65]. RS is also effective against *Xanthomonas oryzae* pv. *oryzae*, which causes bacterial blight in rice. The inhibition is attributed to oxidative stress induced by RS, as well as disruption of energy, purine, amino acid, and NAD+ metabolism, due to the presence of double bonds and hydroxyl groups in its structure [66].

In addition to its direct antimicrobial activity, RS can also serve as a precursor for the synthesis of potent antimicrobial oligomeric stilbenes through oxidative coupling using silver acetate (AgOAc). These compounds have exhibited antifungal activity against *Plasmopara viticola* and *B. cinerea* [67]. In addition, no significant negative effects on soil microbial communities have been observed in studies of genetically modified rice producing resveratrol, indicating little impact on the environment [68].

**p-Coumaric acid (p-CO)** has demonstrated significant antifungal properties, particularly against *B. cinerea* and *Penicillium expansum*. It notably inhibits the mycelial growth of these fungi and reduces the production of patulin, a toxic metabolite from *P. expansum*. These results suggest that p-CO enhances the antioxidant capacity and defense responses of sweet cherry fruit to fungal pathogens [69]. Furthermore, p-CO is a key component of grape marc, and hydrolysates from grape marc have shown promising antifungal activity against *F. oxysporum* and *Alternaria* spp., positioning it as a potential safe alternative to conventional antifungal agents in agriculture [70].

It was also confirmed that a p-coumaric acid formulation does not negatively affect soil bacterial communities. In a study by Kalwasińska et al. [71], neither the p-CO formulation nor the synthetic fungicide Porter 250 EC (with the active compound difenoconazole) significantly affected bacterial biodiversity or nitrogen-cycle bacteria. However, both formulations altered bacterial community structure. The p-CO-based formulation caused more changes in bacterial phylotype abundance after two weeks, but fewer after four weeks, suggesting potential for bacterial homeostasis restoration and compatibility with existing agricultural practices. p-Coumaric acid has the ability to inhibit the growth of *A. alternata, B. cinerea*, *F. culmorum, F. oxysporum*, and *F. solani*. In in vitro tests against these pathogens, the fungicide Porter 250 EC showed stronger antifungal activity at lower concentrations, while in seed contamination tests on rapeseed, cabbage, and cucumber, p-CO was as effective as Porter 250 EC [72]. In addition, p-coumaric acid can accumulate in the rhizosphere, resulting in soil acidification [73].

**Gallic acid (GA)** has demonstrated significant antifungal and antibacterial activities. In vitro and in pot experiments, GA inhibited the growth of *Alternaria solani*, without inducing any phytotoxic effects on tomato plants [74]. Additionally, GA proved effective against *B. cinerea*, a common fungal pathogen in tomato crops. Low-molecular-weight chitosan–GA conjugates reduced fungal fruit rot by 83% [75]. GA and its derivatives also exhibit antifungal effects when present in plant and algae extracts. For instance, a methanolic extract from *Cinnamomum camphora* containing GA showed antifungal activity against *A. alternata*, *Fusarium solani*, and *F. oxysporum*. At a concentration of 4000 µg/mL, it inhibited the fungal mycelial weight by 60%, 49%, and 24%, respectively [76].

Furthermore, GA has shown efficacy against bacterial plant pathogens that typically require the use of harmful cupric salts for control. In a study by Francesconi et al. [77], a nanostructured formulation containing cellulose nanocrystals, high amylose starch, chitosan, and GA at 0.05% (*w*/*v*) concentration exhibited both in vitro and in vivo biocidal activity against *Pseudomonas syringae* pv. *actinidiae* (Psa), *Pseudomonas syringae* pv. *tomato* (Pst), and *Pseudomonas savastanoi* pv. *savastanoi* (Psav), offering a safer alternative to traditional antimicrobial agents.

**Tannic acid (TA)** has shown effective antifungal properties against various pathogens. A 1% (*w*/*v*) TA solution inhibited conidia germination and mycelial growth of *Fusarium graminearum* by 75–80%. In climate chamber experiments with the wheat variety Apogee, treatment with a 5% (*w*/*v*) TA suspension reduced deoxynivalenol (DON) mycotoxin levels by 81%, while field trials with commercial wheat varieties showed an average DON reduction of 66%. Notably, TA applications during flowering sometimes performed better than synthetic fungicides [78].

TA also effectively targets *P. digitatum*, responsible for citrus green mold. In vitro, it inhibited mycelial growth and spore germination, and in vivo tests on artificially inoculated citrus fruits demonstrated a 70% reduction in disease severity, primarily through disruption of the fungal cell wall and plasma membrane [79]. Moreover, TA has been incorporated into advanced formulations to enhance its efficacy. For instance, TA-modified pro-ethyl cellulose nanoparticles improved the stability and sustained release of active compounds against *F. graminearum* [80]. Similarly, a nanomicrocapsule system combining a rosin-based triazole derivative with TA enhanced water solubility, leaf adhesion, and antifungal activity against *Rhizoctonia solani*, maintaining effectiveness over an extended period with low toxicity [81]. In addition to its antimicrobial activity, tannic acid can serve as a soil remediation agent by reducing metal toxicity, which also supports its use in sustainable agriculture [82].

**Ferulic acid (FA)** has demonstrated significant antifungal properties across various plant pathogens. FA showed strong activity against *B. cinerea*, including fungicide-resistant isolates, and exhibited promising results in combating infections in injured and contaminated grapes [83]. In tomato, treatment with 100 μM FA enhanced resistance to *B. cinerea* by reducing disease incidence and lesion area. This increased resistance was linked to the activation of the salicylic acid and jasmonic acid signaling pathways, along with an upregulation of nitric oxide synthase, which boosted nitric oxide levels [84].

FA also contributed to the detoxification of *F. graminearum* mycotoxin deoxynivalenol (DON) when combined with 365 nm light irradiation. This photoreaction led to the degradation or modification of DON, presenting a potential alternative method for controlling fungal contaminants in agriculture and environmental water [85].

**Salicylic acid (SA)** has shown significant antifungal activity across various plant pathogens. Methanolic extracts from *Thompson seedless* grape, *Ziziphus*, pomegranate, and fig were found to contain SA, which exhibited antifungal effects in vitro against *A. solani*, *B. cinerea*, and *Botrytis fabae* [86]. A study confirmed that SA effectively inhibits the growth of *B. cinerea* in vitro, with similar results observed for its derivatives, methyl salicylic acid, and acetylsalicylic acid. Proteomics analyses revealed that SA and methyl salicylic acid affected both intracellular and extracellular proteomes, suggesting mechanisms such as pH regulation, metal homeostasis, mitochondrial respiration, ROS accumulation, and cell wall remodeling to explain the observed fungal growth inhibition [87].

In another study, SA, in combination with chitosan, reduced the germination percentage of *Botrytis*, *Penicillium*, and *Alternaria* species isolated from blueberries by 90%. Application of SA at 5 mM also decreased the incidence of phytopathogens in stored blueberries, suggesting that SA can serve as an alternative to synthetic fungicides for controlling fungal pathogens in fruit storage [88]. Additionally, a study exploring the synergistic effects of SA and endophytic fungi (*Aspergillus oryzae* and *Aspergillus tubingensis*) to control Fusarium wilt in tomatoes demonstrated that this combination improved plant fitness, enhanced photosynthetic pigments, and boosted antioxidant enzyme activity [89].

**Eugenol (EU)** has been extensively studied for its antifungal, antibacterial, and antiviral properties. It exhibits antifungal activity against a wide range of pathogens, including *A. niger*, *Aspergillus terreus*, *B. cinerea*, *Monilinia fructigena*, *Penicillium* species, *Phytophthora nicotianae*, *F. graminearum*, and *Fusarium avenaceum* [38,90,91,92,93]. Additionally, EU has been shown to possess antibacterial activity against *Salmonella* sp. and antiviral effects against tomato yellow leaf curl virus [94,95].

In combination with other compounds like thymol (TH), carvacrol (CR), and cinnamaldehyde (CN), EU demonstrates enhanced antimicrobial effects. A study by Yang et al. [96] observed that the antifungal rates of CR, TH, and EU reached 100% at 400 mg/L, with EC50 values of 43.40, 56.22, and 86.63 mg/L, respectively, for carvacrol, thymol, and eugenol against *S. sclerotiorum*. EU also showed potential in managing bacterial wilt disease caused by *R. solanacearum* when combined with TH [97].

**Thymol (TH)** exhibits strong antimicrobial properties, particularly when combined with other phenolic compounds. In a study by Ji et al. [98], soil treatments with TH significantly reduced bacterial wilt in tomatoes caused by *R. solanacearum.* Another study explored a TH-based nanoemulsion for its antibacterial effects, showing substantial in vitro growth inhibition of *Xanthomonas axonopodis* pv. *glycine* in soybeans [99]. Additionally, TH-loaded chitosan nanoparticles (TCNPs) demonstrated antibacterial activity by inhibiting biofilm formation and the production of exopolysaccharides and xanthomonadin in *Xanthomonas campestris* pv. *campestris* [100].

The antifungal activity of TH has been extensively researched. In one study, TH-derived submicron nanoemulsion effectively inhibited *F. graminearum*, the pathogen responsible for Fusarium head blight (FHB) in wheat [101]. TH also enhanced the fungicidal effects of Tebuconazole and Difenoconazole against various phytopathogens, including *Bipolaris sorokiniana*, *Parastagonospora nodorum*, and other fungi like *Fusarium* spp. and *Alternaria* spp. [102]. Moreover, TH nanoemulsions showed efficacy in controlling *B. cinerea*, the cause of postharvest gray mold on tomato fruit [103].

TH is also effective in preventing aflatoxin accumulation during grain storage, although its high volatility limits its use. To address this, a TH–betaine co-crystal system was synthesized, which exhibited enhanced thermal stability and effectively inhibited the growth of *A. flavus* and the production of aflatoxin B1 [104]. Additionally, in a study by Kmoch et al. [63], TH, along with other essential oils like cinnamaldehyde and carvacrol, showed strong antifungal effects against *H. solani*, the cause of silver scurf in potatoes, offering a sustainable alternative for pathogen control during storage.

### 4.2. In Food Packaging

Phenolic compounds can be used to improve food safety and extend freshness and shelf life by reducing microbial growth. Due to the antimicrobial properties of phenolic substances, they have potential applications in the food industry, including as a component of active packaging materials. This approach is particularly relevant in the context of contamination by food-borne pathogens such as *Escherichia coli*, *Listeria monocytogenes*, *Salmonella* spp., *Shigella* spp., *Campylobacter* spp., or *S. aureus*, as well as mold fungi, including *Aspergillus* spp. or *Penicillium* spp. [105].

Phenolic substances can be introduced into packaging in various forms. It can be coating the inner surface of the packaging with active compounds, which is a good solution for substances that are immiscible with polymer. A second solution is to immobilize active compounds on polymers by means of ions or covalent bonds, which requires the presence of a functional group in the compound and in the polymer. Another approach involves placing sachets of active compounds in food packaging, on a similar basis to oxygen or moisture absorbers. In contrast, the most common type of active-packaging creation involves direct incorporation of the compound into the polymer, which allows for even distribution of the substance in the package and slow release [106].

**Cinnamaldehyde (CN)** can be effectively used as an antimicrobial agent in food packaging systems designed to extend shelf life and reduce the risk of foodborne illnesses. The substance is “generally recognized as safe” (GRAS) and has no mutagenic effects [107]. In nanoencapsulated form and as an additive to polylactide (PLA), CN has demonstrated significant antimicrobial activity against *Escherichia coli* W1485 and *B. cereus* ATCC 14579 [108]. Cinnamaldehyde also has significant antifungal activity, including against *P. expansum* at concentrations of 1.5 to 5% (g/100 g protein) and *A. niger* at concentrations of 3 and 5% (g/100 g protein). As a result, it can be used as an additive to packaging materials to extend the shelf life of food products, including bread and cheese [109].

CN is useful for protecting porous foods such as bread because it is a volatile compound. Therefore, it acts throughout the package, not just where the food comes into contact with the film. PLA and poly(butylene adipate-co-terephthalate) (PBAT) films with CN added at concentrations of 2–10% (*w*/*w*) have been shown to extend the shelf life of bread by 21 days by inhibiting the growth of *Penicillium* sp. and *A. niger* [110]. The addition of CN to the packaging also extends the freshness of other food products, including extending the freshness of raw beef by 4 days, due to its antimicrobial activity against *Staphylococcus aureus* and *E. coli* [111], and strawberries, by inhibiting the growth of mold, for up to 15 days [112].

**Cinnamic acid (CA)** can be successfully incorporated into food packaging materials to achieve antimicrobial effects. It is a compound that is considered safe, with no genotoxic effects [113]. One of the materials developed was a film based on sodium alginate and pectin. It was a material that biodegraded about 43% in 15 days in soil and also showed antimicrobial activity against various species of foodborne bacteria. A fresh beef packaging trial also confirmed a reduction in bacterial contamination of about 84% and improved organoleptic properties [114]. Another material was starch- and PLA-based films with surface-applied CA, which had an inhibitory effect on the growth of *E. coli* and *Listeria innocua*. This study showed higher efficacy of PLA/starch/PLA trilayer materials than monolayer materials. Also, applying the active ingredient by electrospinning was more effective than spraying the solution [115]. Films made from cassava starch with cinnamic acid also confirmed antibacterial activity against *E. coli* and *L. innocua*, both in tests on microbial media and on fresh chicken breast and melon [116]. Another approach to extending the shelf life of foods is to use antimicrobials not as packaging additives but by directly applying them to foods. A nanoemulsion with trans-CA showed MIC values of 0.78 mg/mL and 3.13 mg/mL for *S. aureus* and *P. aeruginosa*, respectively, and the results indicated that when used on fresh-cut lettuce, it could serve as a natural preservative [117].

**Carvacrol (CR)** can also be successfully utilized in the food industry. It is considered a safe food additive. Safety in humans was confirmed in a phase I clinical study in which subjects were treated with 1 mg/kg/day or 2 mg/kg/day [118]. It can be added to baked goods, chewing gum, and non-alcoholic beverages. Another approach is incorporating carvacrol into packaging materials. In a study by López-Mata et al. [119], chitosan films with added carvacrol at concentrations of 0.5%, 1%, and 1.5% (*v*/*v*) were produced. It was demonstrated that the 1.5% (*v*/*v*) concentration exhibited antibacterial activity against *S. typhimurium* and *E. coli* O157:H7. There are many other reports in the literature about the antimicrobial properties of chitosan films with added carvacrol [120,121,122,123]. Enriched films are also being developed, including biocidal chitosan–cyclodextrin films [124] and chitosan–pullulan films [125]. CA also exhibited an inhibitory effect on *E. coli* O157:H7, when applied in synergism with medium-chain fatty acids (MFCAs). The combined treatment could overcome the disadvantages of MCFAs such as unpleasant odor and high cost because the required concentrations can be reduced indicating that it could be successfully used to eliminate food-borne pathogens, significantly improving the microbiological safety of foods [126].

Also, cellulose acetate films with carvacrol at concentrations of 10% by weight showed antibacterial activity against *Weissella viridescens* and *Pseudomonas fluorescens*. Additionally, a trial involving the packaging of pork ham in this material showed that the packaging extended the freshness of the meat by 2.8 times compared to the control sample [127].

Carvacrol has been incorporated into various polymer matrices, including polypropylene [128], the commercially available Mater-Bi^®^ matrix [129], gelatin [130], soy protein isolate [131], poly(vinyl alcohol) [132], polylactic acid [133], polylactic acid/polybutylene adipate terephthalate [134], starch [135], starch/polyester [136], and polyethylene [137].

**Resveratrol (RS)** also has potential applications in the packaging industry due to its antimicrobial and antioxidant properties. Given its natural occurrence in fruits such as grapes, its use in the food industry raises no concerns, especially since this compound also exhibits anticancer properties [15]. Busolo and Lagaron [138] developed polyethylene films with resveratrol and montmorillonite clay, demonstrating strong antioxidant and antimicrobial effects of the tested films against *S. aureus* CECT 86T and ATCC 12600. Additionally, they reported a low level of resveratrol migration from the films into water (<0.01 mg/kg) and confirmed the absence of migration into isooctane. Resveratrol was also encapsulated in gelatin/zein fiber mats [139]. The material exhibits antimicrobial properties against *E. coli* and *S. aureus.* Furthermore, pieces of raw pork were packaged in the material, showing that the packaging could extend the shelf life of the meat by 3 days.

RS may also be useful in active packaging of raw poultry to control *Campylobacter*. Cellulose derivative materials (hydroxyethylcellulose and cellulose acetate) with resveratrol showed antimicrobial activity against *C. coli* 873, *C. coli* ATCC 33559, *C. jejuni* 225421, and *C. jejuni* ATCC 33560 [140]. In another study, RS and its encapsulated form in methylated-β-cyclodextrin showed activity against *Campylobacter* spp., with MICs ranging from 64 to 512 μg/mL. However, the encapsulated resveratrol was characterized by 400-fold better solubility in water, and it showed no cytotoxic effects [141].

**p-Coumaric acid (p-CO)** can also be used in the food industry as a food preservative by inhibiting the growth of food spoilage bacteria. It occurs naturally in many plant foods, so it is considered a safe substance. It is characterized by low toxicity in mouse models. Recent studies even point to its antipyretic or anticancer effects [16]. Chitosan (CH) derivatives grafted with CO showed a broad spectrum of antibacterial activity, including against *S. aureus* and *P. aeruginosa* [142]. Chitosan coatings are well suited as packaging for soft fruits, including strawberries. Chitosan films with p-coumaric acid produced by Liu et al. [143] showed strong antimicrobial activity against *S. aureus* and were slightly less effective in inhibiting *E. coli*, confirming the greater susceptibility of Gram-positive bacteria to phenolic compounds. In addition, the coatings exhibited antifungal activity against *B. cinerea* and apparently prolonged the shelf life of the strawberries packed in them. In the case of polyvinyl alcohol/starch composite films, the addition of p-coumaric acid resulted in antimicrobial properties against *E. coli* and *S. aureus*, in addition to reducing the film’s cytotoxicity [144]. Another interesting approach is electrospun zein fibers modified with the addition of p-CO and caffeic acid. The addition of these compounds improved the mechanical properties of the fibers, which could potentially be used in active food packaging, also showing antimicrobial activity against *E. coli* and *S. aureus* [145].

**Gallic acid (GA)** is commonly found in tea leaves, walnuts, or fruits. It. can also be used to extend the freshness of food as an additive to antibacterial coatings based on various polymers, including chitosan. Chilled meat coated with chitosan and GA extended its shelf life from 6 days to 18 days. The addition of GA also increased the antibacterial properties of chitosan against *Pseudomonas* sp., *Acinetobacter* sp., *Brochothrix thermosphacta, E. coli, S. aureus, Salmonella* sp., and *L. monocytogenes* [146].

GA also has antimicrobial activity against other bacteria, with an MIC of 500 μg/mL for *P. aeruginosa*, 1500 μg/mL for *E. coli*, 1750 μg/mL for *S. aureus*, and 2000 μg/mL for *L. monocytogenes*. The MBC for *E. coli* was 5000 μg/mL, 5250 μg/mL for *S. aureus*, 5500 μg/mL for *L. monocytogenes*, and 500 μg/mL for *P. aeruginosa*. GA led to irreversible changes in membrane properties (charge, intra- and extracellular permeability, and physicochemical properties) through hydrophobicity changes, a decrease in negative surface charge, and the occurrence of local ruptures or pore formation in the cell membranes with consequent leakage of essential intracellular constituents [147].

Also, **tannic acid (TA)** finds an application in food packaging. It is generally recognized as safe (GRAS). When introduced into sodium alginate, it showed antimicrobial activity against *E. coli* and *S. aureus*, directly proportional to the concentration. Moreover, the addition of TA reduced water vapor permeability, i.e., improved the barrier properties of the packaging [148]. In addition to its antimicrobial properties, TA also exhibits protein crosslinking ability, so it can perform both functions in protein-based films, such as gelatin. The ability to crosslink proteins is due to physical and chemical mechanisms, including hydrogen bonding and π-π stacking with the benzene rings in phenylalanine (Phe), tyrosine (Tyr), and tryptophan (Trp), as well as the formation of covalent bonds [149,150]. TA can crosslink not only proteins but also other polymers such as biodegradable poly(butylene adipate-co-terephthalate) (PBAT). It was shown that the film formed from the combination of PBAT, TA, and carbon nanoparticles had good barrier and antimicrobial properties against *S. aureus* and *E. coli* [151].

**Ferulic acid (FA)** is an acceptable food additive, naturally occurring in fruits and vegetables, including tomatoes. It exhibits antimicrobial activity against many foodborne bacteria. As an additive to a polylactide and poly(butylene adipate-co-terephthalate) (PBAT)-based film, it had a lethal effect against *L. monocytogenes* and *E. coli*, with films with 10% FA showing the highest efficacy, which was compared against controls without the active substance [152]. One of the more interesting approaches to creating active food packaging is the creation of composite nanofibers from polyvinyl alcohol, wheat gluten, and glucose, with TA as an antimicrobial additive. The entirety was cross-linked using the Maillard method, and films with 12% (*w*/*w*) FA showed the best biocidal capabilities against *S. aureus* and *E. coli* [153]. As a compound commonly found in plant foods, TA can also be used in edible packaging, including soy protein isolate-based films. In addition to its antimicrobial properties, it has a number of properties that are useful in the food industry. A shelf life extension study of lard indicated that its mechanism of action was to prevent oxidation by reducing the oxygen permeability of the packaging material. In addition, it also acted as a crosslinking agent [154].

There are many reports regarding packaging materials with **salicylic acid (SA)** incorporated. In one paper, starch-based films with SA were tested in vitro, as well as on actual food samples. The latter study, using yogurt, showed that for the first 2 days, there was no difference in bacterial growth in the control sample without SA and in the films without SA. In contrast, extending the storage time of the yogurt to 7 and 10 days resulted in a statistically significant inhibitory effect [155]. The method of introducing SA into a package can affect its properties. Hu et al. [156] showed that chitosan films with SA introduced by the coupling method had better antioxidant, antimicrobial, and barrier properties than incorporated films. In addition, salicylic acid was encapsulated in halloysite and incorporated into alginate or pectin matrices. The alginate films were shown to have a controlled release of salicylic acid in 50% ethanol, which simulated food [157].

**Eugenol (EU)** occurs in the essential oils of many plants and is a safe and non-mutagenic substance. In active food packaging, EU is often used in combination with other antimicrobial substances, including cinnamaldehyde [158] or pediocin [159]. It is also used on its own, for example, in zein- and PLA-based films, where it acts as a plasticizer in addition to its antimicrobial properties. The biocidal properties of the resulting film were similar to the efficacy of pure eugenol against *S. aureus* and *E. coli* at concentrations of 5 and 10% (*v*/*v*) [160]. EU’s effectiveness in extending food shelf life has been demonstrated not only in in vitro tests. Pork was wrapped in yam starch films with EU at a concentration of 26.8 mg/g [161].

**Thymol (TH)** is recognized by regulatory agencies as a safe substance at acceptable levels of consumption. Its antimicrobial properties are due to its ability to damage the cell membrane and increase its permeability. It has the ability to bind to the membrane and induce leakage of intracellular components, causing cell death. It acts on both Gram-positive and Gram-negative bacteria [162]. Although thymol is mainly used in polymeric materials with potential medical applications [163], it can also be an additive to active food packaging, including polypropylene-based [164] or PLA [165]. In PLA-, PBAT-, or poly(butylene succinate) (PBS)-based films, eugenol has been shown to be effective in reducing biofilm formation on the surface of materials, as confirmed on pathogens isolated from dairy products *Bacillus pumilus*, *Bacillus subtilis*, *Bacillus tequilensis*, and *Stenotrophomonas maltophilia* [166].

## 5. Summary

Phenolic compounds of natural origin are gaining increasing attention due to their antimicrobial properties and potential applications in various industries. As presented in this review, phenolic compounds exhibit a variety of antimicrobial mechanisms. An important area of application for phenolic compounds is their incorporation into active food packaging, which extends the shelf life of various food products. In addition, phenolic compounds exhibit antioxidant activity, which further improves the quality and safety of packaged foods.

Despite the potential of phenolic compounds, several challenges remain, including their stability during storage or their release in various applications. Future research should also focus on their environmental impact to ensure sustainable and effective implementation in agriculture and the food industry. The following tables (Table 2 and Table 3) summarize key findings on the antimicrobial activity of selected phenolic compounds and their applications.

## Figures and Tables

**Table 1:** Classification and occurrence of selected phenolic compounds.

Substance	Chemical Name	Group	Occurrence	References
Cinnamaldehyde (CN)	(E)-3-phenyl-2-propenal	Phenylpropanoid	Cinnamon oil (60–75%) from *Cinnamomum cassia* and *Cinnamomum zeylanicum*	[10,11]
Cinnamic acid (CA)	3-phenylprop-2-enoic acid	Phenolic acid (hydroxycinnamic acid)	*Cinnamomum* spp., vegetables, whole grains	[12]
Carvacrol (CR)	2-methyl-5-(1-methylenthyl)-phenol	Monoterpenoid phenol	Essential oils of the Labiatae family, including *Origanum, Satureja, Thymbra, Thymus* and *Corydothymus*	[13,14]
Resveratrol (RS)	3,5,4′-trihydroxystilbene	Stilbene	Various food products, including grapes, red wine, and peanuts	[15]
p-Coumaric acid (CO)	4-hydroxycinnamic acid	Phenolic acid (hydroxycinnamic acid)	Fruits, vegetables and grains	[16]
Gallic acid (GA)	3,4,5-trihydroxybenzoic acid	Phenolic acid (hydroxybenzoic acid)	Oak bark, tea leaves, as well as fruits and walnuts	[17,18]
Tannic acid (TA)	1,2,3,4,6-penta-O-{3,4-dihydroxy-5-[(3,4,5-trihydroxybenzoyl)oxy]benzoyl}-D-glucopyranose	Tannin	Common plants	[19]
Ferulic acid (FA)	4-hydroxy-3-methoxycinnamic acid	Phenolic acid (hydroxycinnamic acid)	Ubiquitous in seeds, and leaves	[20]
Salicylic acid (SA)	2-hydroxybenzoic acid	Phenolic acid (hydroxybenzoic acid)	Willow bark, fruits (berries, grapes), vegetables	[21]
Eugenol (EU)	4-Allyl-2-methoxy phenol	Phenylpropanoid	*Amiaceae*, *Lauraceae*, *Myrtaceae* and *Myristicaceae* families, and in clove oil from *Syzygium aromaticum*	[22]
Thymol (TH)	2-Isopropyl-5-methylphenol	Monoterpenoid phenol	Thyme (*Thymus* spp.)	[23]

**Table 2:** Phenolic compounds of natural origin and their antifungal activity.

Substance	Target Microorganisms	Mechanism of Action	References
Cinnamaldehyde	*Aspergillus* spp., *Fusarium oxysporum*, *Penicillium digitatum*, *Phytophthora capsici*	Increased oxidative stress, cell membrane damage, disrupted fatty acid, polysaccharide and leucine metabolism	[26,29,39,40,42,45]
Cinnamic acid	*Botrytis cinerea*, *Fusarium* spp., *Pyricularia grisea*	Disruption of organelle function, induction of cell death	[12,167]
Carvacrol	*Fusarium oxysporum*, *Cladosporium* spp., *Alternaria alternata*	Damage to cell membrane, leakage of intracellular components	[58,168]
Resveratrol	*Ralstonia solanacearum*, *Xanthomonas oryzae*	Damage to bacterial membrane, inhibition of metabolism	[65,66]
Eugenol	*Aspergillus* spp., *Penicillium* spp.	Destruction of cell membrane, inhibition of protein synthesis	[90,91]

**Table 3:** The use of phenolic substances in active food packaging.

Substance	Polymer Matrix	Impact on Food Shelf Life	Antimicrobial Effect	References
Cinnamaldehyde	Polylactide (PLA), sodium alginate	Extend bread freshness by 21 days, protect raw beef and strawberries	Inhibition of growth of *Penicillium* spp., *Aspergillus niger*, *E. coli*, *S. aureus*	[110,111,112]
Cinnamic acid	Sodium alginate and pectin starch, PLA	Reduction of bacterial contamination of raw beef by 84%	Antibacterial activity against *E. coli* and *L. innocua*	[114,115,116]
Carvacrol	Cellulose acetate	Extend freshness of pork ham 2.8 times	Antimicrobial activity against *Weissella viridescens* and *Pseudomonas fluorescens*	[127]
Resveratrol	Poliethylene (PE)	Delay fat oxidation, extend meat shelf life	Antimicrobial activity against *S. aureus*, *Campylobacter* spp.	[137,140]
Eugenol	Zein, PLA	Increase film flexibility, extend pork shelf life	Inhibiting the growth of *E. coli* and *S. aureus*	[160]

## Data Availability

No new data were created or analyzed in this study. Data sharing is not applicable to this article.

## Abbreviations

The following abbreviations are used in this manuscript:

CN	Cinnamaldehyde
CA	Cinnamic acid
CR	Carvacrol
RS	Resveratrol
CO	p-Coumaric acid
GA	Gallic acid
TA	Tannic acid
FA	Ferulic acid
SA	Salicylic acid
EU	Eugenol
TH	Thymol
ROS	Reactive oxygen species
MIC	Minimum inhibitory concentration
DON	Deoxynivalenol
PLA	Polylactide
PBAT	Poly(butylene adipate-co-terephthalate)
MFCAs	Medium-chain fatty acids
CH	Chitosan
PBS	Poly(butylene succinate)
